# Prioritizing US Cervical Cancer Prevention With Results From a Geospatial Model

**DOI:** 10.1200/JGO.2015.001677

**Published:** 2016-04-11

**Authors:** Jonathan K. Kish, Alicia I. Rolin, Zhaohui Zou, James E. Cucinelli, Zaria Tatalovich, Mona Saraiya, Sean F. Altekruse

**Affiliations:** **Jonathan K. Kish**, **Alicia I. Rolin**, **Zaria Tatalovich**, and **Sean F. Altekruse**, National Cancer Institute, Rockville; **Zhaohui Zou** and **James E. Cucinelli**, Information Management Services, Silver Spring, MD; and **Mona Saraiya**, Centers for Disease Control and Prevention, Atlanta, GA.

## Abstract

**Purpose:**

To determine if differences in screening and vaccination patterns across the population may accentuate ethnic and geographic variation in future burden of disease.

**Methods:**

Using Cancer in North America data provided by the North American Association of Central Cancer Registries, county cervical cancer incidence trends from 1995 to 2009 were modeled for the entire United States using ecologic covariates. Rates for health service areas were also modeled by ethnicity. State-level incidence was mapped together with Papanicolaou (Pap) screening, past 3 years (women ≥ 18 years old), and three-dose human papillomavirus (HPV) vaccine coverage (girls 13 to 17 years old) to identify potential priority areas for preventive services.

**Results:**

US cervical cancer incidence decreased more during the periods 1995 to 1999 and 2000 to 2004 than during the period 2005 to 2009. During these 15 years, the most affected areas became increasingly confined to Appalachia, the lower Mississippi Valley, the Deep South, Texas, and Florida. Hispanic and black women experienced a higher incidence of cervical cancer than both white and Asian and Pacific Islander women during each period. Women in 10 of 17 states/districts with a high incidence (≥ 8.14/100,000) reported low Pap testing (< 78.5%), HPV vaccine coverage (< 33.9%), or both prevention technologies.

**Conclusion:**

The decline in cervical cancer incidence has slowed in recent years. Access to HPV vaccination, targeted screening, and treatment in affected populations is needed to reduce cervical cancer disparities in the future.

## INTRODUCTION

Cervical cancer incidence and mortality in the United States have declined steadily in the decades since the adoption of cytology-based Papanicolaou (Pap) testing.^1^ Recently, cervical cancer death rates seem to have plateaued, and a slight decrease has been reported in the use of Pap testing.^2^ Cervical cancer disparities (the uneven distribution of burden to areas of lower socioeconomic status and minority ethnic groups) are increasing.^3-6^ Cervical cancer incidence is elevated among older and black women^7,8^ and in impoverished areas such as Appalachia, the lower Mississippi Valley, and the United States–Mexico border area.^9^ Reduced access to screening services is a driving factor in these areas. Nationally, 3-year Pap screening completion is > 80% for women 21 to 65 years of age, with lower levels of screening among immigrants, minorities, the poor, and older women.^10^ Approximately 47% of girls born in the United States in 2000 had received at least one dose of human papillomavirus (HPV) vaccine by age 13 years.^11^ Safety concerns and views on sexual activity influence parental decisions regarding the vaccination of adolescent girls.^11^ Although the safety and efficacy of the HPV vaccination is well established, in 2013, 38% of girls 13 to 17 years of age in the United States had completed the three-dose HPV vaccine series,^11^ far less than optimal.^12^ Because the reduction in cervical cancer incidence as a result of HPV vaccination will take decades to be realized,^12^ surveillance is needed to identify communities with a higher burden of cervical cancer and to direct cervical cancer screening and vaccination services where they are needed most.^1^

To define high-priority areas for preventive services, we estimated small-area cervical cancer incidence, including by ethnicity, and mapped state-level incidence with percentage screening and HPV vaccination coverage.

## METHODS

### Incidence Data

The North American Association of Central Cancer Registries (NAACCR) provided a Cancer in North America data set with county-level incidence and population denominators for this analysis. A total of 36 of 50 state registries, all of which met NAACCR silver or gold standards for tumor registration data quality, gave active consent to include their incident cases in the analytic data set. For the years 1995 to 2009, complete or partial county-level cervical cancer incidence data were reported by NAACCR registries in areas covering 74% of the United States population. This included complete data from 1995 to 2009 for 22 states encompassing 56% of the population (Arizona, California, Colorado, Connecticut, Hawaii, Idaho, Illinois, Iowa, Kentucky, Louisiana, Maine, Michigan, Nebraska, New Jersey, New Mexico, New York, Pennsylvania, Rhode Island, Texas, Utah, Washington, and Wyoming) and multiple years of data for 14 states encompassing 18% of the population (Alaska, Arkansas, Georgia, Massachusetts, Mississippi, Montana, Nevada, North Carolina, North Dakota, South Carolina, South Dakota, Tennessee, Virginia, and West Virginia). In these 14 states, data were missing for a median of 2 reporting years, generally the earliest reporting years. Two lower Mississippi Valley states were missing data for 1995 to 2003. The remaining 14 states and the District of Columbia, which did not provide data, accounted for 26% of the US population.

### Spatiotemporal Model

We developed a model similar in composition to one used commonly to predict current-year cancer incidence in the United States^13^ to estimate county-level cervical cancer incidence in 5-year intervals from 1995 to 2009.^14^ The multivariable logistic regression model included a set of covariates selected through a forward and backward process. A generalized linear model was used (PROC GLIMMIX, SAS 9.3, SAS Institute, Cary, NC) with three random terms to account for spatial autocorrelation (longitude and latitude of the county), temporal autocorrelation (year of diagnosis), and residual autocorrelation of covariates. Missing cervical cancer incidence at the county level was modeled on the basis of the reported incidence in counties with comparable attributes. The incidence for counties with observed data was also updated according to model predictions to slightly adjust reported rates. The time periods of interest for modeled all-ethnicity county-level incidence were 1995 to 1999, 2000 to 2004, and 2005 to 2009. To present incidence among ethnic groups, data were aggregated at the health service area (HSA) level,^15^ reducing instability from small counts at the county level. HSAs are either a single county or a cluster of contiguous counties that are relatively self-contained with respect to hospital care. Data were suppressed when there were 16 or fewer modeled incident cases. The accuracy of predicted rates for areas with missing data depends on how well covariates in the model predict the actual incidence.

### Model Inputs

Demographic inputs of cervical incident cases were non-Hispanic ethnicity (hereafter referred to as white, black, American Indian and Alaska Native, and Asian and Pacific Islander), Hispanic ethnicity (all ethnicities), and age. County of residence was geocoded on the basis of latitude and longitude. County-level population estimates were obtained from the US Census Bureau Summary File for each year from 1995 through 2009. Incidence data, stratified by age (< 44, 45 to 64, and ≥ 65 years) and year of diagnosis were retrieved using SEER*Stat 8.1.2 (Information Management Services, Calverton, MD).

County-level covariates included in the model were county-level rural-urban density data,^16^ an Area Health Resources covariate enumerating the number of hospital-based physicians at the county level,^15^ and data on the percentages of the county population who were black, Asian and Pacific Islander, and American Indian and Alaskan Native. Cervical cancer mortality data reported by the National Center for Health Statistics were also included for each county in the United States. Socioeconomic covariates incorporated into the model were the percentage of the county population with income below the poverty level and the percentage of the population ≥ 25 years of age with ≥ 4 years of college education. The model also contained a variable indicating whether the county was in a National Program of Cancer Registries–funded area. The model provided estimates of county-level cervical cancer incidence for the entire United States, including areas and years with missing data as well as those with reported data. To illustrate geographic distributions in rates, modeled county estimates were mapped using 2000 Census county designations (ArcGIS 10.1; ESRI, Redlands, CA). In the all ethnicities combined model uncertainty related to small numbers was addressed with spatial smoothing. The population-weighted, nonparametric algorithm used universal Kriging after detrending.^17^ Data were approximately normally distributed and no transformations were applied, although first-order surface trend was removed. Smoothing was not applied to incidence maps of specific ethnic groups because of the potential for instability.

### State-Level Incidence Trends

State-level modeled incidence trends on the basis of estimated age-standardized incidence were analyzed using joinpoint regression analysis (Joinpoint 3.5.0; Information Management Services). The technique fit a series of joined straight lines on a logarithmic scale for annual age-standardized rates.^18^ Using data from the model for each year, state trends were estimated for 5-year fixed intervals, or annual average percent change (AAPC), using weighted annual percent changes from joinpoint models.

### Identification of Priority Areas

Three state-level variables were visualized: modeled cervical cancer incidence during the period 2005 to 2009, state-level proportions of women 21 to 65 years of age who had had a Pap test during the past 3 years^19^ estimated from pooled 2008 to 2010 responses to the National Health Interview Survey and Behavioral Risk Factor Surveillance System, and HPV vaccination coverage by state (obtained from the National Immunization Survey of Teens performed during 2013)^20^ defined as the percentage of girls 13 to 17 years old who had received three doses or more of either the bivalent or the quadrivalent vaccine.

State-level cervical cancer incidence was mapped in tertiles (low, midlevel, and high incidence). Low incidence was 5.17 to 6.75, midlevel incidence ranged from 6.76 to 8.13, and high incidence was 8.14 to 9.76 cases per 100,000 women. Low state-level Pap screening coverage was defined as < 78.5%, midlevel as 78.5% to 81.4%, and high as 81.5% to 88.5%. Low state-level HPV vaccination uptake was defined as < 33.9%, midlevel as 33.9% to 40.1%, and high as 40.2% to 56.5%. States in the bottom third distribution for three-dose HPV vaccine receipt (< 33.9%), Pap screening (< 78.5%), or both vaccine and Pap screening were depicted with horizontal, vertical, and crosshatched lines, respectively. The status of state Medicaid expansion as of January 2016 was also assessed.^21^

## RESULTS

Modeled cervical cancer incidence and observed death rates per 100,000 women from 1995 to 2009 are presented in Table 1. Incidence and death rates were highest among non-Hispanic black followed by Hispanic women in each time period. Incidence and death rates decreased from the period 1995 to 1999 to the period 2005 to 2009 among all ethnic groups. Age-specific incidence per 100,000 women was highest among 45- to 64-year-old women, averaging 17.3 and decreasing from 20.5 during the period 1995 to 1999 to 15.2 during the period 2005 to 2009. The highest death rate was also seen among women ≥ 65 years of age, averaging 7.0 and decreasing from 8.2 during the period 1995 to 1999 to 6.1 during the period 2005 to 2009.

**Table 1:**
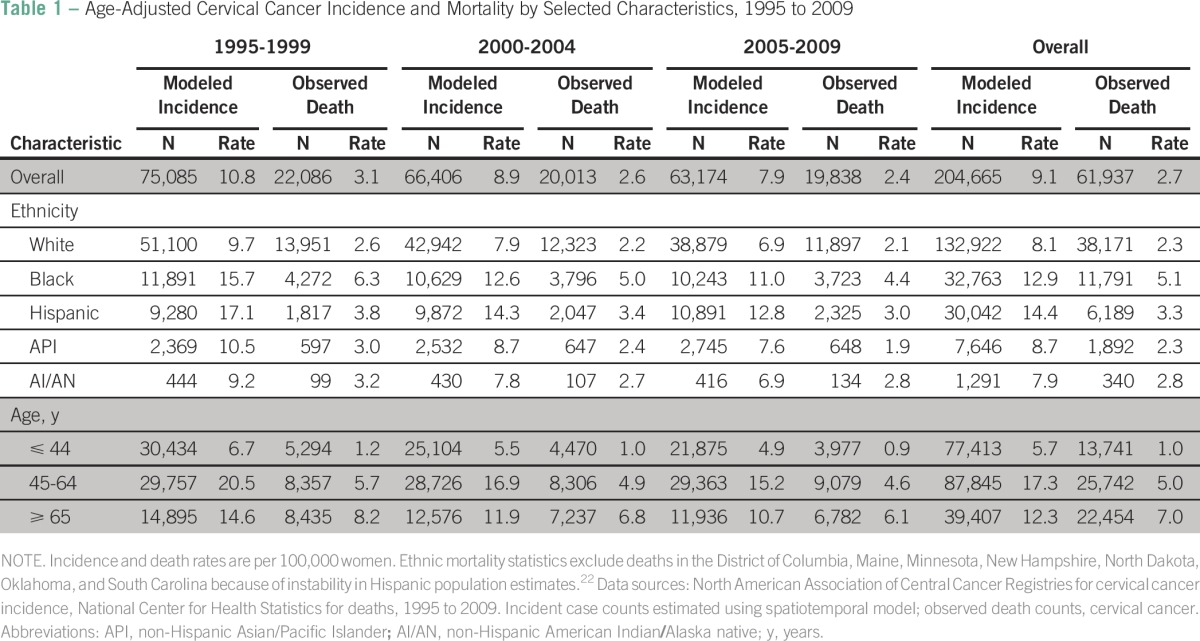
Age-Adjusted Cervical Cancer Incidence and Mortality by Selected Characteristics, 1995 to 2009

**Fig 1:**
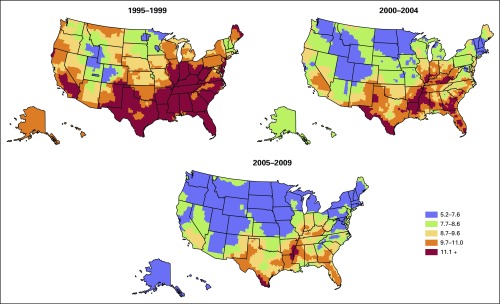
Smoothed, county-level modeled US cervical cancer incidence per 100,000 women, 5-year intervals, 1995 to 2009.

Figure 1 displays the smoothed, county-level modeled estimates of cervical cancer incidence by time period. From 1995 to 1999, the highest incidence was found in counties extending south and west from Appalachia to southeastern Colorado, northeastern New Mexico, and Texas, with a low incidence found in southwestern New England, the Northern Plains, and Mountain West. During the period 2000 to 2004, elevated rates became more localized to central Appalachia, the Ohio River Valley, the Lower Mississippi River Valley, rural Texas including the Mexican border area, and the rural Southeast. During the period 2005 to 2009, areas with the highest incidence were even more contained within Appalachia, the lower Mississippi Valley, the Deep South, Texas, and Florida.

Overall, in the United States during 1995 to 1999, 2000 to 2004, and 2005 to 2009, the AAPC in modeled cervical cancer incidence declined significantly by −3.5, −3.1, and −1.1 percent per year over successive periods (Data Supplement). This pattern of slowing but still statistically significant decreasing incidence trends in recent years was seen in 27 states. In nine states and in the District of Columbia, trends did not change significantly over the three time periods. In three states (Georgia, Virginia, and Louisiana) there was a nonsignificant increase in cervical cancer incidence between 2005 and 2009. In nine other states (Alaska, Tennessee, Maryland, New Jersey, South Carolina, South Dakota, Illinois, Iowa, and Oregon), the decrease in cervical cancer incidence during the period 2005 to 2009 was not statistically significant. In Utah and Oklahoma the AAPC during the period 1995 to 1999 was not statistically significant; however, incidence decreased significantly during more recent time periods.

**Fig 2:**
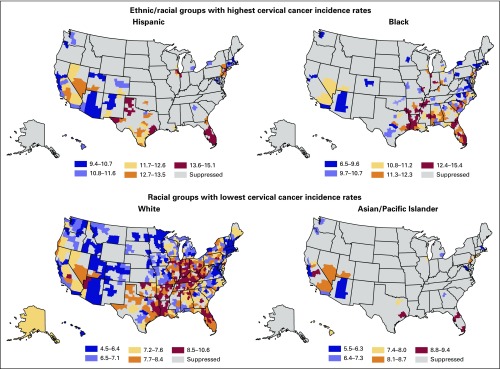
Modeled health services area–level cervical cancer incidence by ethnicity, 2005 to 2009.

Figure 2 presents modeled HSA-level incidence by ethnicity during the period 2005 to 2009. Color ranges vary by map. Higher cervical cancer incidence was seen among Hispanic and black than among white and Asian and Pacific Islander women. Among whites, incidence was highest in Appalachia, the Mississippi and Ohio River valleys, Indiana, and rural parts of Illinois, Texas, Louisiana, Arkansas, Oklahoma, Tennessee, Alabama, and Georgia. Isolated areas with high incidence were found in central California, northwestern Arizona, central Pennsylvania, southern New Jersey, and northern and central Florida. High modeled incidence areas for blacks were found in eastern Texas and adjacent areas of Oklahoma, Louisiana, and Arkansas. The incidence was also elevated along the Mississippi River Valley from Illinois to Tennessee, Arkansas, and Mississippi. Affected rural areas included adjacent areas of Mississippi and Alabama, and Georgia and north central Florida. A high incidence was also seen in northern and central Indiana, rural South Carolina, southern New Jersey, New York City, and southeastern Florida.

Of areas with large Hispanic populations, the regions with the highest modeled incidence included west Texas, central and southern Florida, and metropolitan Houston, Chicago, and New York. Among Asian and Pacific Islanders, a high modeled incidence was seen in central California and Houston, Texas; central and south Florida; and metropolitan New York. The incidence for American Indians/Alaska Natives was not presented because of low counts.

**Fig 3:**
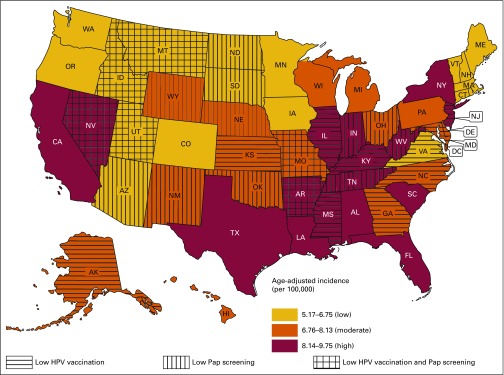
States with high, midlevel, and low cervical cancer incidence during the period 2005 to 2009, further depicting states with low percentages of Papanicolaou (PAP) screening or human papillomavirus (HPV) vaccination. Less than 78.5% of women > 18 years old reported PAP screening in the past 3 years during the period 2008 to 2010, and < 33.9% of girls 13 to 17 years of age had received three doses of HPV vaccine as of 2013.

Figure 3 presents state-level modeled cervical cancer incidence during the period 2005 to 2009. States are classified into three categories: low, midlevel, and high cervical cancer incidence states. States in the bottom tertile of Pap screening use and HPV vaccine uptake were classified as having low adoption of these preventive services. Sixteen states plus the District of Columbia, shown in red, had a high cervical cancer incidence. In two high-incidence states, both Pap screening and HPV vaccine uptake were low (Arkansas and Nevada). There was a low proportion of Pap screening in three high-incidence states (Tennessee, West Virginia, and Indiana) and of HPV vaccine uptake in five high-incidence jurisdictions (District of Columbia, Mississippi, Illinois, Kentucky, and New Jersey). Many of the 16 states with a midlevel cervical cancer incidence (shown in orange) were adjacent to high-incidence states. One midlevel-incidence state (Missouri) had low uptake of both Pap screening and HPV vaccination. Among the midlevel-incidence states, five (New Mexico, Wyoming, Nebraska, Oklahoma, and Ohio) had a low percentage of Pap screening, and five (Alaska, Kansas, Maryland, North Carolina, and Georgia) had low vaccine uptake. States with the lowest cervical cancer incidence (shown in gold) were located in New England, the northern tier of states, the four corners region, and Virginia. In three low-incidence states (Montana, Idaho, and Utah), a low percentage of women received Pap screening, and a low percentage of girls received three doses of HPV vaccine. There was also a low percentage of Pap screening in Arizona, North Dakota, and South Dakota, and the percentage of girls in Virginia who received HPV vaccination was less than 33.9%.

As of January 2016, six of 17 jurisdictions with the highest cervical cancer incidence had not expanded Medicaid coverage (Alabama, Florida, Mississippi, South Carolina, Tennessee, and Texas). Seven of 16 states with a midlevel cervical cancer incidence had not expanded Medicaid (Georgia, Kansas, Missouri, North Carolina, Nebraska, Oklahoma, and Wyoming). Among the 18 states with the lowest cervical cancer incidence, three had not expanded Medicaid (Idaho, Montana, and Utah).

## DISCUSSION

This study suggests that the long-term decrease in US cervical cancer incidence is slowing down. This finding is consistent with the nonsignificant decreasing trend reported during the period 2005 to 2009 in areas in the United States with high-quality incidence data.^12^ High-incidence geographic areas were confined increasingly to rural areas within Appalachia, Texas, the lower Mississippi Valley, and the southeastern United States. Hispanic and black women had a higher cervical cancer incidence than did white and Asian and Pacific Islander women. In 10 of 17 states/districts with a high cervical cancer incidence, there was low use of Pap screening or HPV vaccination. Locally tailored cervical cancer vaccination, screening, and treatment efforts that target poor women living in medically underserved geographic areas are needed to maintain progress in reducing cervical cancer disparities.

As recently as the 1970s, cervical cancer was a leading cause of cancer among US women; however, the incidence has decreased in subsequent decades.^8^ Trends in this study differ from sustained decreasing cervical cancer incidence trends over the past half-century, but are consistent with recent findings describing a leveling off of cervical cancer mortality^2^ and incidence^12^ in the United States. A possible explanation for the slowing of the decreasing trend is that women with access to health care are benefiting from preventive services such as Pap screening and HPV testing,^2^ to a greater extent than are women in medically underserved groups. These underserved women, who experience a higher burden of cervical cancer,^23^ include ethnic minorities, women from low socioeconomic backgrounds,^5^ and women living in impoverished geographic areas.^10^ Future progress to reduce the burden of cervical cancer depends on access to vaccination, screening, and treatment of these hard-to-reach groups.^24^ Of note, some states with the highest incidence of cervical cancer have low percentages of Pap screening and HPV vaccine uptake. Provisions of the Affordable Care Act, which require most health insurance plans to cover cervical cancer screening and HPV vaccination with no cost sharing, could improve cervical cancer prevention among low-income women.^25^

In this study spanning the years 1995 through 2009, there were progressively smaller areas with an elevated cervical cancer incidence over time. Regions with the highest burden of disease during the period 2005 to 2009 were largely contained to economically deprived counties within Appalachia, Texas, the lower Mississippi Valley, and the southeastern United States. The limited progress in reducing the incidence of this cancer in areas with slow economic development or an influx of immigrant populations is consistent with findings reported in Mexico,^26^ Brazil,^27^ and England.^28^

Lack of awareness, lack of access to health care, and cultural beliefs are barriers to cervical cancer prevention within population subgroups.^29-31^ For instance, in Connecticut,^32^ heterogeneity in the occurrence of cervical cancer precursors is reported. Culturally competent locally targeted outreach needs to be part of cervical cancer control programs. One study of African-American women living in the high cervical cancer mortality area of Sunflower County, Mississippi, indicated that door-to-door visits to offer home self-collection HPV test kits increased participation in cervical cancer screening almost four-fold compared with clinic-based Pap testing alone.^33^ In a national study, predictors of not being screened for cervical cancer included not having made a physician office visit within the past 12 months because of cost, minority ethnicity, lack of a high school diploma among residents of metropolitan areas, and self-reported fair or poor general health among nonmetropolitan area residents.^34^ The heterogeneity of underserved women suggests a need for screening and HPV vaccination outreach across broad areas.^35^

Other researchers have reported ethnic and geographic disparities in cervical cancer screening^34^ and incidence.^36^ In the United States–Mexico border area, Hispanic women were less likely than other women to have had a recent Pap test,^37^ and white women in Appalachia had higher rates of HPV infection compared with the US population.^38^ Although provider recommendation improves acceptance of HPV vaccination, minority and low-income women are least likely to receive such recommendations.^39^ Cervical cancer prevention can be advanced through community-based interventions,^40^ particularly in communities with limited access to a formal health care system. These community-based interventions may be more effective than one-size-fits-all approaches.^41-43^

This study identified populations that would benefit from cervical cancer outreach by ethnicity, geography, and access to screening and HPV vaccination. Ongoing spatial analysis is recommended to monitor cervical cancer trends in the HPV vaccine era.^44^ Study limitations include missing data for some states, which was partially addressed with geospatial modeling. Furthermore, HPV vaccination data were available only at the state level. County-level data would improve prioritization of outreach to areas with a high cervical cancer burden. Despite limitations, the analysis identifies priority areas for interventions to improve screening and vaccination rates. Although progress has been made in reducing the incidence of cervical cancer, outreach is needed in low-socioeconomic areas of the United States. Provisions of the Affordable Care Act that eliminate cost sharing for cervical cancer screening and HPV vaccination in most health plans should reduce cost as a barrier to receiving these prevention services.
